# Al-induced proteomics changes in tomato plants over-expressing a glyoxalase I gene

**DOI:** 10.1038/s41438-020-0264-x

**Published:** 2020-04-01

**Authors:** Xudong Sun, Hui Li, Santosh Thapa, Sasikiran Reddy Sangireddy, Xiaobo Pei, Wei Liu, Yuping Jiang, Shaolan Yang, Dafeng Hui, Sarabjit Bhatti, Suping Zhou, Yong Yang, Tara Fish, Theodore W. Thannhauser

**Affiliations:** 1 Department of Agricultural and Environmental Sciences, College of Agriculture, Tennessee State University, 3500 John A Merritt Blvd, Nashville, TN 37209 USA; 2 College of Horticulture, Shandong Agricultural University, Taian, Shandong P.R. China; 3 R.W. Holley Center for Agriculture and Health, USDA-ARS, Cornell University, Ithaca, NY 14853 USA

**Keywords:** Abiotic, Protein-protein interaction networks

## Abstract

Glyoxalase I (Gly I) is the first enzyme in the glutathionine-dependent glyoxalase pathway for detoxification of methylglyoxal (MG) under stress conditions. Transgenic tomato ‘Money Maker’ plants overexpressing tomato *SlGlyI* gene (tomato unigene accession SGN-U582631/Solyc09g082120.3.1) were generated and homozygous lines were obtained after four generations of self-pollination. In this study, *SlGlyI-*overepxressing line (GlyI), wild type (WT, negative control) and plants transformed with empty vector (ECtr, positive control), were subjected to Al-treatment by growing in Magnavaca’s nutrient solution (pH 4.5) supplemented with 20 µM Al^3+^ ion activity. After 30 days of treatments, the fresh and dry weight of shoots and roots of plants from Al-treated conditions decreased significantly compared to the non-treated conditions for all the three lines. When compared across the three lines, root fresh and dry weight of GlyI was significant higher than WT and ECtr, whereas there was no difference in shoot tissues. The basal 5 mm root-tips of GlyI plants expressed a significantly higher level of glyoxalase activity under both non-Al-treated and Al-treated conditions compared to the two control lines. Under Al-treated condition, there was a significant increase in MG content in ECtr and WT lines, but not in GlyI line. Quantitative proteomics analysis using tandem mass tags mass spectrometry identified 4080 quantifiable proteins and 201 Al-induced differentially expressed proteins (DEPs) in root-tip tissues from GlyI, and 4273 proteins and 230 DEPs from ECtr. The Al-down-regulated DEPs were classified into molecular pathways of gene transcription, RNA splicing and protein biosynthesis in both GlyI and ECtr lines. The Al-induced DEPs in GlyI associated with tolerance to Al^3+^ and MG toxicity are involved in callose degradation, cell wall components (xylan acetylation and pectin degradation), oxidative stress (antioxidants) and turnover of Al-damaged epidermal cells, repair of damaged DNA, epigenetics, gene transcription, and protein translation. A protein–protein association network was constructed to aid the selection of proteins in the same pathway but differentially regulated in GlyI or ECtr lines. Proteomics data are available via ProteomeXchange with identifiers PXD009456 under project title ‘25Dec2017_Suping_XSexp2_ITAG3.2’ for *SlGlyI-*overexpressing tomato plants and PXD009848 under project title ‘25Dec2017_Suping_XSexp3_ITAG3.2’ for positive control ECtr line transformed with empty vector.

## Introduction

Soil acidification and the associated Al^3+^ toxicity are abiotic stress factors threatening agriculture and food security worldwide. Increasing areas of forest and agricultural land are becoming acidic (regionally reported as low as pH < 5.5, 4.0, 3.0), caused by long-term over-application of nitrogen (N) and sulfur (S) fertilizers, acid rain (NO_3_, SO_4_, CO_2_), and other environmental factors^1–5^. In these soils, most agricultural plants experience stresses from metal phytotoxicity (Al, Fe, Mn) and reduced availability of essential nutrients, such as P, Ca, Mg, K, leading to low or no yield^1,2,6,7^.

Plants when exposed to suboptimal conditions including toxic levels of Al^3+^, will rapidly overproduce methylglyoxal (MG)^8–13^. Excessive levels of MG cause DNA mutation and glycation of proteins^14–16^. The impairment of protein function exacerbates oxidative stress, and leads to activation of deleterious signal transduction pathways^17^. A study on mung bean (*Vigna radiata* L. cv. BARI Mung-2) showed that Al-treatment-induced overproduction of MG in seedlings, and exogenous application of spermine induced an increase in glutathione (GSH) pool and Gly II activity to ameliorate the injurious effects of MG^10^.

Two systems are used in plants to detoxify MG. The first is the GSH-dependent glyoxalase pathway; the second pathway involves glyoxalase enzyme Gly III (DJ-1), which converts MG to d-lactate in a single step without the intermediate processes^18^. In the GSH-dependent glyoxalase pathway, Gly I is the first enzyme which catalyzes the isomerization of MG into S-2-hydroxyacylglutathione and then Gly II hydrolyzes these thiolesters to produce d-lactate, which will eventually be converted into pyruvate to feed into tricarboxylic acid (TCA) cycle^19–21^. Since its initial characterization of the glyoxalase pathway back in 1913^22,23^, the importance of the glyoxalase system in plant defense against various types of biotic and abiotic stresses has been more and more firmly recognized^24,25^.

Tomato (*Solanum lycopersicum*) plants grow best at pH 6.0–6.8, and they are sensitive to salt, acid soil (pH < 5.0), and Al^3+^ toxicity^3,26–28^. Previous studies have reported that in tomato the gene expression and enzyme activity of glyoxalase I (Gly I) are induced when plants respond to salt and osmotic stresses as well as phytohormonal stimuli^25,29^. Transgenic tomato ‘Ailsa Craig’ overexpressing glyoxalase I (*GlyI*) and glyoxalase II (*GlyII*) genes became more tolerant to high salt level (800 mM NaCl), as these plants were protected from lipid peroxidation and production of H_2_O_2_ in leaf tissues under the stress condition^30^. These studies provided evidence for the role of the GSH-dependent glyoxalase system in conferring tolerance to abiotic stress factors in tomato plants.

In a proteomics analysis of Al-treated tomato ‘Money Maker’, a lactoylglutathione lyase/Gly I was identified as a differentially expressed protein^31^. In this study, transgenic tomato plants overexpressing the respective gene were generated, and the function of the gene in conferring Al tolerance was evaluated. Quantitative proteomics-based approach has been widely used to reveal dynamics of protein changes during various biological processes^32–34^. We have used the tandem mass tags (TMT) and isobaric tags for relative and absolute quantitation (iTRAQ) proteomics in studies of stress proteomes in tomato and switchgrass^28,35,36^. In this study, the Al-induced proteomes were identified using the TMT-proteomics approach. Functional classification analysis of quantified proteins was performed to help understand the biological processes associated with the function of the *GlyI* gene. A protein association network map was constructed to visualize protein changes within and across functional clusters and pathways.

## Materials and methods

### Preparation of *SlGlyI* constructs, and generation of transgenic plants

A tomato cDNA clone LEFL-1034BD04 (tomato unigene accession SGN-U582631 or Solyc09g082120.3.1) was obtained from Kazusa DNA Research Institute, Tokyo, Japan. The insert sequence was confirmed by re-sequencing the plasmid. The open-reading frame (ORF) region of *GlyI* was amplified using polymerase chain reaction (PCR) from the cDNA clone with the addition of BamH1 and XhoI restriction sites at the 5 and 3 termini. The PCR product was cloned onto pSAT-RNAi vector driven by 2 × 35S promoter^37^. The expression cassette (2 × 35S promoter-*GlyI*-terminator) on the pSAT-RNAi vector was isolated with (PI-PSPI) enzymes and sub-cloned onto the pRCS2-ocs-bar binary vector. The *GlyI* construct and the empty vector (pRCS2-ocs-bar binary vector) were then transferred into *Agrobacterium tumefaciens* strain LBA4404 by electroporation, which were then used for tomato transformation^38^.

### Genetic transformation

Tomato transformation followed the procedure decribed previously with minor modification^39,40^. Tomato **‘**Money Maker’ seeds were sterilized in 50% bleach for 30 min with agitation and followed by three rinses in sterile water. Seeds were germinated on solidified agar plates containing 1/2 strength Murashige and Skoog (MS) basal medium (Sigma, St. Louis, MO, USA) and maintained at 26 °C under a 16/8 h (d/n) photoperiod. Excised cotyledons from 6 to 8-d-old seedlings with both ends cut were soaked in *A. tumefaciens* harboring the binary construct cultures (overnight culture in liquid TY medium plus 40 mg/L acetosyringone, OD600 of 0.1–0.2) for ~10 min. Inoculated explants were then cultured with abaxial side facing down on MS basal medium supplemented with 2.0 mg/L zeatin riboside, and 40 mg/L acetosyringone and incubated at 23 °C for 2–3 d. Shoots were induced on MS plate supplemented with 2.0 mg/L zeatin riboside (ZR), 500 mg/L carbenicillin plus 4 mg/L glufosinate, and ZR concentration was reduced to 0.5 mg/L for shoot elongation. Rooting was done on Gamborg’s B5 basal medium (Sigma), supplemented with 0.2 mg/L indole 3-butyric acid (IBA) and 500 mg/L carbenicillin plus 4 mg/L glufosinate^41^. Plants transformed with *GlyI* construct were referred as GlyI lines, and those with the empty vector as ECtr lines. Ten individual transformation events were selected for GlyI and ECtr lines each. Rooted plants were potted in Miracle-Gro potting mix in two gallon pots (7 L) in a greenhouse. Tomato plants were self-pollinated and each plant was allowed to grow two tomatoes and additional fruits were removed.

To select for homozygous transgenic lines, seeds from the same fruit of T1 plants were re-potted. Germinating seedlings at the two cotyledon stage were sprayed with 0.2% dl-phosphinothricin ammonium salt (Sigma) twice a day for 2 weeks. The wild type seeds were grown as negative controls, all of which died within 1 week of the herbicide treatments. The ratio of living/dead seedlings was used to predict the copy number of insertions in each transgenic line. Plants with a ratio of 3:1 were taken as evidence of containing a single copy insertion of transgenes. Living plants were grown to maturity. Seeds from these plants were tested for herbicide resistance following the same procedure as in T1. In T2 generation, plants producing 100% herbicide resistance seedlings were considered as homozygous lines; these plants were propagated in T3 generations to confirm the homozygosity. Transgenic plants were further validated by PCR with 35S forward and gene-specific reverse primers for *SlGly* and primers for the *bar* gene (35SF: CTATCCTTCGCAAGACCCTTC, *GlyI*R: TAGGAGGCAGGAGCCCCA; *bar*F-5′-AGTCGACCGTGTACGTCTCC-3′, *bar*R-3′-GAAGTCCAGCTGCCAGAAAC-5′). The PCR bands were cloned onto T/A cloning vector and plasmids were sequenced to confirm that the *SlGlyI* and *bar* genes were inserted in the tomato genome (Fig. S1). The positive transgenic plants were grown for an additional generation (T4) and offspring plants were validated using the same PCR procedure. Seeds derived from an individual plant of GlyI and ECtr (the positive control) of T4 generation, and non-transgenic wild type (WT) plants were harvested, and used as experimental materials in Al treatment experiments described below.

### Al treatments and plant measurments for phenotyping

Seeds were submerged in 10% bleach for 10 min with constant shaking, followed by three rinses in ultra-pure water. Seeds were sown in vermiculite on fiberglass screen mesh (wire diameter of 0.013 inch = 0.33 mm) in plastic net pots (6 inch = 15.24 cm) which were inserted into 1-m tall hydroponic PVC tubes (Supplemental material Fig. S1). The tubes were filled with Magnavaca’s nutrient solution (pH 4.5)^42^. When seedlings grew to first-true leaf stage, two plants of similar sizes were transplanted into one tube. In a week, plants grew out new leaves, when they were thinned to a single plant per tube.

The Al treatment experiments began when tomato plants grew to the size of bearing three true leaves. For the Al-treated groups, the basic solution was supplemented with 100 µM AlK_2_SO_4_·12H_2_O providing 20 µM Al^3+^ ion activity. The non-treated control groups were refreshed with the basic nutrient solution only. Each line had three biological replicates each comprising 10 plants. Experimental plants were planted following a completely randomized design. The treatment solutions were refreshed weekly, and acidity of the solution was maintained at pH 4.5–5.0 by testing using a pH strip (pH 3.5–5.5) (Fisher Scientific, CA, USA)^28^. After 30 d of treatments, plants were carefully removed from the vermiculite in the net-pots. The length, fresh weight of shoot, and root were recorded for each individual plant. Dry weight was taken after the tissues were dried at 70 °C in an oven until constant weight. For the detection of Al accumulation on root-tips, the distal 3 cm of primary and lateral roots were dissected for the tomato plants followed by submerging in hematoxylin solution for 30 min^43,44^. The Al-treatment experiments were conducted in a greenhouse where the temperature was maintained at 25 ± 2 °C, without supplemental light.

### Al treatments for proteomics analysis

The proteomics analysis was conducted on GlyI and ECtr lines. As these analyses require a large number of root-tips for protein extraction, each pot/tube was seeded with 20–30 plants. The Al treatment was applied directly in the same tube without the transplanting step. Three biological replicates each comprising of three pots/tubes were included for each treatment condition. The Al treatment followed the same procedure as in the phenotyping experiments.

### Root tip tissue harvest

For root-tip tissue harvest, the distal 5 mm root tips were cut using a surgical blade from roots grown through the fiberglass mesh in net pots. Tissues were either frozen immediately in liquid N_2_ and stored at −80 °C for proteomics analysis, or stored in prechilled extraction buffer for enzyme and MG extraction.

### MG content and Gly I activity assay

Immediately after being excised from plants, root tips were transferred to a pre-chilled protein extraction buffer containing 0.1 M phosphate-buffered saline (PBS) (pH 7.4), 0.1% Triton, and 1X Halt protease inhibitor cocktail (ThermoFisher, CA, USA). Tissues were homogenized with the lysing matrix (frozen to −20 °C before use) from FastDNA™ Spin kit (MP Biochemicals, CA, USA), using a MM 400 Mill Mixer (Retsch GmbH, Germany) which was run at 30/s frequency and 40 s each cycle for five cycles. After centrifugation at 14,000×*g*, 4 °C for 10 min, supernatant containing protein was collected. Protein concentration was determined following the instruction in the Qubit protein assay kit (Fisher Scientific) using a Qubit 3.0 Fluorometer (Life Technologies Corporation, NY, USA)^36,44^. Glyoxalase activity was assayed by following the formation of S-lactoylGSH (SLG) from adducts of MG and GSH which is catalyzed by Gly I, using the Gly I activity assay kit (BioVision, Milpitas, CA, USA)^45^. The absorbance at OD240 was recorded every 20 s for up to 20 min at 26 °C with orbital intervals shaking (2 s between each read), using a SpectraMax M5 (Molecular Devices, San Jose, CA, USA). The absorbance at different time points in the linear range (delta OD) was used to calculate enzyme activity.

MG extraction was conducted following the method described previously^46^ with modifications. Immediately upon dissection from plants, the distal 5 mm root tip tissues were rinsed three times in double-distilled (dd) H_2_O. For the extraction of MG, samples were homogenized in 5% perchloric acid (1:1; w/v) and centrifuged at 13,000×*g* for 10 min at 4 °C. After centrifugation at 12,000×*g* for 1 min, supernatant was transferred to a spin module with filter, and centrifuged at 14,000×*g* for additional 5 min. The obtained flow throw was used for estimating MG following the manufacturer’s instruction using a MG assay kit (Biovision)^45^. The reduced chromophore product was recorded at 450 nm in end-point mode using SpectraMax M5 (Molecular Devices).

### Protein extraction for proteomics analysis

Protein extraction followed a method described previously^36,47,48^. Frozen root tissue was ground to a fine powder under liquid nitrogen. The powered tissue was mixed with acetone containing 10% trichloroacetic acid (TCA) (2:10; w:v) followed by incubation at −20 °C overnight. After centrifugation at 16,000×*g* for 20 min at 4 °C, pellets were collected. Protein pellets were then washed in 80% methanol containing 0.1 M ammonium acetate and 80% acetone, and finally dried under vaccum to allow complete evaporation of acetone and moisture. Protein was purified from pellets using a modified SDS/phenol extraction method. For TMT labeling, protein was solubilized in a buffer containing 500 mM triethylammonium bicarbonate (TEAB), 0.1% sodium dodecyl sulfate (SDS), 8 M urea, and 1X protease inhibitors (Sigma). To be compatible with the TMT-labeling reaction, the urea concentration was reduced to 1 M, TEAB to 100 mM by adding appropriate volume of 50 mM TEAB containing protease inhibitors^28,36,47,48^. Protein concentration was determined by using a Bradford protein assay kit (Bio-Rad, Hercules, CA, USA).

### TMT labeling and mass spectrometry analysis of root-tip proteins

Protein samples were reduced using tris (2-carboxyethyl) phosphine hydrochloride and the resulting thiols were alkylated with iodoacetamide. The proteins were then isolated by precipitation using six volumes of cold acetone (−20 °C) for 12 h. The pellets were isolated by centrifugation at 4 °C and 16,000×*g* for 10 min. Excess acetone was removed by decanting, and the pellets were air dried at 4 °C.

Each pellet containing 100 µg protein was reconstituted in 100 mM TEAB. Digestion was initiated by the addition of 2.5 µg of modified, sequencing grade, porcine trypsin (Promega, Madison, WI) and allowed to continue for 12 h at 32 °C. The tryptic peptides were labeled using 10-plex TMT (ThermoFisher) according to the instructions of the manufacturer. For the GlyI line, the three Al-treated replicates were labeled with tags 127C, 129N, 130C and non-Al treated control replicates labeled with 126, 128N, 129C; and for the ECtr plants, the Al-treated samples were labeled with tags 128N, 129C, 131, and non-treated control samples labeled with 126,128C, 130N. Each labeling reaction was allowed to proceed for 1 h and was terminated by the addition of an excess of hydroxylamine (8 µl of a 5% solution) to scavenge the unreacted labeling reagent.

The appropriatelly labeled samples from each experiment were pooled together. SDS, scavenged labels and other labeling reaction by-products were removed by solid phase extraction using C18 Sep-Pak cartridges (Waters, Milford, MA, USA). Bound peptides were eluted in 0.5 ml of the elution solvent containing 50% (v/v) acetonitrile (ACN) in 0.1% aqueous trifluroacetic acid (TFA). The eluted samples were dried under reduced pressure in a CentriVac Concentrator (LabConCo, Kansas City, MO, USA)^28,36,47,48^.

The TMT-tagged tryptic peptides were reconstituted in 20 mM ammonium formate, pH 9.5, and loaded onto an XTerra MS C18 column (3.5 μm, 2.1 × 150 mm, Waters) with 20 mM ammonium formate (NH_4_FA, pH 9.5) as buffer A and 80% ACN/20% 20 mM NH_4_FA as buffer B. The chromatography was carried out on a Dionex UltiMate 3000 system using a gradient from 10% to 45% buffer B in 30 min at a flow rate of 200 μL/min. Forty-eight fractions were collected at 1 min intervals. The fractions were pooled using a multiple fraction concatenation strategy^48,49^ into a total of 12 samples. All of the samples were dried and reconstituted in 100 μL of 2% ACN/0.5% FA for nanoLC–MS/MS analysis.

Nano-scale liquid chromatography (LC)–MS/MS was carried out on an Orbitrap Fusion (ThermoFisher) trybrid mass spectrometer equipped with an UltiMate3000 RSL nano-LC system (Dionex) using a method similar to those described previously^44,50,51^. Briefly, each of the 12 peptide fractions were injected on a PepMap C-18 trap column (3 µm, 75 µm × 2.0 cm, Dionex) at 20 µL/min to remove salts and other interfering compounds. Bound peptides were then eluted from the trap column and separated on a PepMap C-18 nano column (3 µm, 75 µm × 15.0 cm, Dionex) at 300 nL/min using a linear gradient of 5–38% ACN in 0.1% FA.

The mass spectrometer was operated in the positive ion mode with the spray voltage set at 1.6 kV and source temperature at 275 °C. The quadrupole, ion trap, and FT were calibrated using the polysiloxane ion at *m*/*z* 445.120025 as an internal calibrant. The experiment employed a data-dependent analysis strategy (DDA) using the FT mass analyzer to acquire one high-resolution survey scan followed by a 3 s “top speed” HCD-MS/MS acquisition at 37.5% normalized collision energy to fragment multiply charged precursor ions above an ion count threshold of 10^4^. The survey scans were carried out at high resolution (120,000 fwhm) for the range 400–1600*m*/*z*. Settings for AGC and MaxIT were 1e5 and 120 ms, respectively. The Q isolation window was set at 1.6*m*/*z* for the range 105–2000*m*/*z*. Excalibur 3.0 and Tune 2.0 (ThermoFisher) were used to acquire all data.

For data analysis, Proteome Discoverer 2.2 (ThermoFisher) was used to search the experimental data against the tomato protein database ITAG 3.20. The search criteria were as follows: proteolytic enzyme, trypsin, two missed cleavages allowed; fixed modifications were set to the S-carbamidomethylation of cysteine and TMT modification of peptide terminal and lysine ε amines; the variable modification allowed included the oxidation of methionine and the deamidation of asparagine glutamine. Precursor ion mass tolerance was set to 10 ppm and fragment ion mass tolerance was 50 mmu. For quantification, the TMT 10-plex method within PD2.2 was used with peptide confidence set at “high”. All quantified proteins are required to include a minimum of two unique peptides. All quantified peptides are required to have reporter ions from all relevant TMT tags. The mass spectrometry proteomics data have been deposited in the ProteomeXchange Consortium via the PRIDE partner repository with the dataset identifiers PXD009456 under project title ‘25Dec2017_Suping_XSexp2_ITAG3.2’ for GlyI line and PXD009848 under project title **“**25Dec2017_Suping_XSexp3_ITAG3.2” for ECtr line (http://www.ebi.ac.uk/pride).

### Global quantification of Al-induced proteomes and identification of Al-induced differentially expressed proteins

For protein quantification, it was required that a protein contains a minimum of two unique peptides which were quantified for all the labled samples in a TMT experiment^44,52^. For GlyI and ECtr lines, the quantifiable proteins were listed first and the adundance ratio (treated/control) of these proteins in PD2.2 report was log2 tranformed. To identify Al-induced differentially expressed proteins (DEPs), the log_2_Fold values, hereafter referred as Fold (T/C), were fit to a normal distribution to obtain the standard deviation (SD) of the quantified proteome. The DEPs for each individual tomato line were listed using the following criteria: Fold (T/C) >2SD (±), *P* < 0.05 values which were determined using a post hoc Tukey honestly significant difference (HSD) test in PD2.2 report, and protein quantified with a minimum of two unique peptides^44,52^.

### Functional analysis and protein association network

The Al-induced proteomes and DEPs were classified into different categories of gene ontology (GOs): biological processes and molecular functions, using Plant MetGenMap^53^. The protein names of Al-induced DEPs were submitted to the Search Tool for the Retrieval of Interacting Genes (STRING) software (v11.0)^54^. Protein–protein interactions were identified through comparing the input data with the S*olanum lycopersicum* annotated genome in the STRING database (https://string-db.org/). Protein clusters were created using the Markov Cluster Algorithm (MCL) inflation parameter (MCL = 3), and the association networks showing protein quantitative changes was visualized in Cytoscape^44,55,56^.

### Statistical analysis

The levels of significant differences were analyzed using ANOVA followed by Fishers least significant difference (LSD) test using SAS software (Version 9.4; Institute Inc., Cary, NC, USA, 2014).

## Results

### Selection and physiological evaluation of GlyI transgenic lines overexpressing the *SlGlyI* gene

**Fig. 1:**
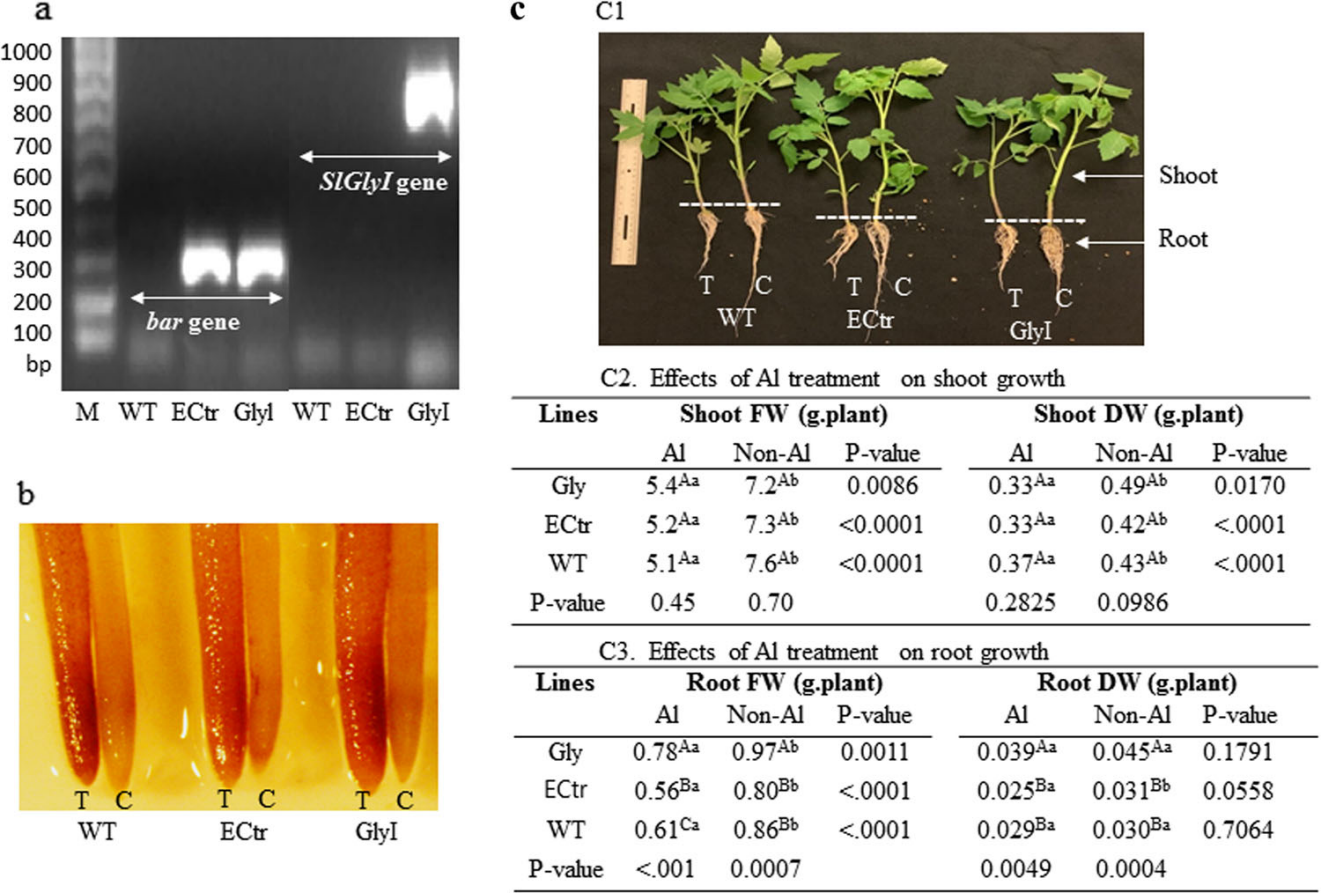
Validation of transgenic plants and phenotypic changes under Al-treated conditions. **a** Polymerase chain reactions (PCR)-amplified DNA fragments for the *bar* gene and *SlGlyI* gene using genomic DNAs from GlyI (tomato line overexpressing *SlGlyI*), ECtr (tomato line transformed with empty vector expressing *bar* as selective marker gene), and WT (wild-type) non-transgenic plants. **b** Hematoxylin-stained roots (T: Al-treated plants; C: non-Al-treated plants). The images were taken in bright field under an Olympus stereo-microscope. **c** Plant growth. C1 Images of plants under Al-treated (T) and non-Al-treated (C) conditions; C2 Shoot growth; and C3 Root growth. The fresh and dry weight data were tested for the levels of significant differences using ANOVA followed by Fishers least significant difference (LSD) test using SAS. Data followed by lower case letters indicate comparison between Al-treated and non-treated conditions within each line, and the capital letters indicate comparison across the three lines. The same letters indicate no significant difference, and different letters indicate significant difference at *P* < 0.05

Using 35S promoter forward primer and *SlGlyI* gene-specific reverse primer, DNA bands matching the size of the insert region were amplified from the *SlGlyI-*overexpressing lines (GlyI), but not in the positive control (ECtr) line, nor the negative control nontransgenic wild type (WT) plants. The *bar* gene fragment was amplified in the GlyI and ECtr, but not in the WT plants (Fig. 1a). These DNA fragments were sequenced which confirmed that they are identical to the insert sequences in the transformation constructs (Fig. S1).

Plants from the three lines were grown in a hydroponic system under Al-treated and non-Al-treated conditions for 30 d. Roots were taken for hematoxylin staining of Al accumulation on root-tips (Fig. 1b). The Al-treated root-tips were stained red but not the non-Al-treated roots, furthermore the staining intensity appeared similar when compared across the three lines. These results indicate that the amount of Al deposited on tomato roots was not influenced by the *SlGlyI* transgenic events.

Plants were divided into shoot and root sections which were measured and processed separately (Fig. 1c1). When compared within each individual line, fresh and dry weight of shoots and roots decreased significantly (*P* < 0.05) under Al-treated condition compared to the non-Al-treated control groups (Fig. 1c2, c3). When compared among the three lines, there were no significant differences in shoot fresh and dry weight (Fig. 1c2). The GlyI plants produced bigger roots (based on fresh and dry weight) than ECtr and WI lines under non-Al treated and Al-treated conditions (Fig. 1c3). When measured by the Al-induced relative decrease in root fresh weight [(Al-Non-Al)/Al*100], the GlyI line had a 7.8% decrease which was lower than ECtr (24%) and WT (25%).

**Fig. 2:**
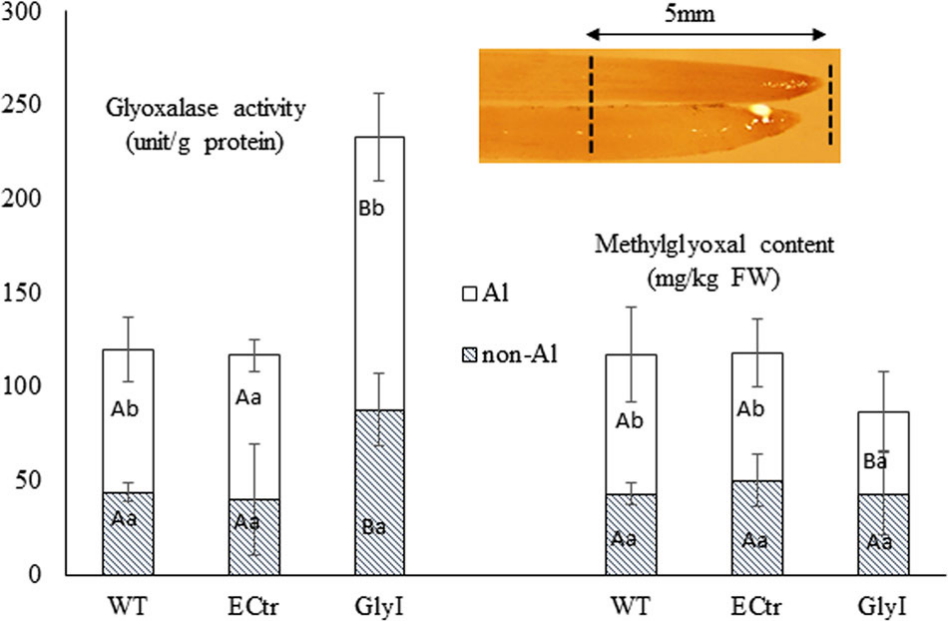
Glyoxalase activity and methylglyoxal content in the basal 0.5 cm root-tips of GlyI overexpressing a SIGlyI gene, positive control ECtr line transformed with empty vector and non-transgenic wild type (WT) plants. For each assay, the lower case letters indicate the comparison between Al-treated and non-Al-treated conditions within each line, and the capital letters indicate comparison across the three lines. The same letters indicate no significant difference, and different letters indicate significant difference at *P* < 0.05. The levels of significant differences were analyzed using ANOVA followed by Fisher’s least significant difference (LSD) test using SAS software. The image shows the basal 5 mm root tip section tissues used in enzymatic and methylglyoxal assays

Root-tips are the major target of Al toxicity, therefore the distal 5 mm root regions (below the maturation region where root hair starts to develop) were taken to measure MG content and Gly I activity. As shown in Fig. 2, when compared among the three lines, the GlyI plants contained a significantly higher level of Gly I activity under both Al-treated and non-Al-treated conditions compared to ECtr and WT lines (*P* < 0.05, represented by capital letters). When compared within each individual line, Gly I activity increased from non-Al-treated control to Al-treated conditions; it reached a significan level in GlyI and WT lines (*P* < 0.05, represented by lower case letters), but not in the ECtr line due to a higher SD in the treatment groups. For the MG content, there was no significant difference among the three lines under non-Al-treated condition. Under Al-treated condition, MG content increased signficantly in ECtr and WT, but not in GlyI. Taken together, these results indicate that the GlyI line was able to regulate the MG level due to the higher level of Gly I activity compared to the other two lines under Al-treated condition.

### Global quantification of Al-induced proteomes

**Fig. 3:**
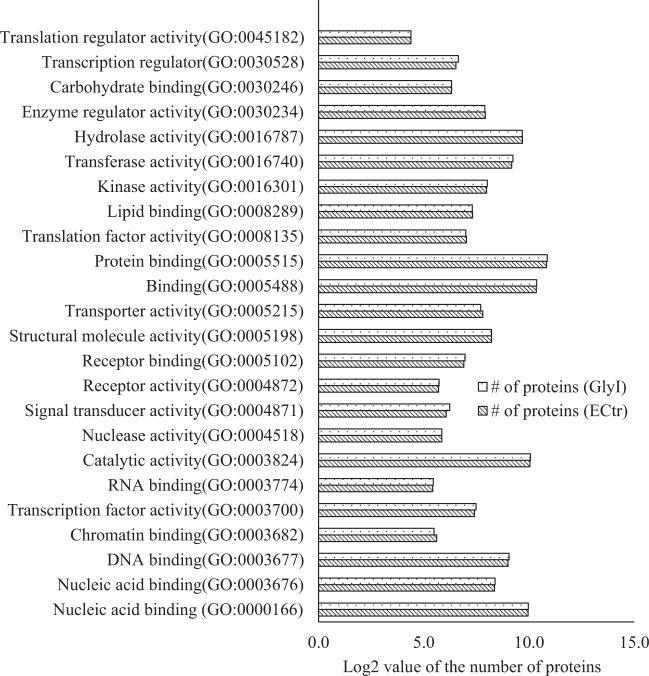
GO term classification of molecular functions of GlyI (A) and ECtr (B) lines classified by Plant MetGenMap. Analysis was performed using the 4024 quantified proteins in GlyI and 4184 proteins in ECtr lines. Data are given as the log2-transformed number of proteins in each GO term. Several quantified proteins were not found in the annotated tomato genome database in Plant MetGenMap

In this study, the TMT-based quantitative proteomics experiments were used to identify the Al-induced proteomes in GlyI and ECtr lines. In GlyI line, 4080 quantifiable proteins were identified, and 4273 proteins were identified in ECtr line (Tables S1 and S2). According to the molecular functional classification using the Plant MetGenMap system, these proteins were classified into 24 gene ontology (GOs) terms and an unidentified group (Fig. 3, Table S3). These proteins are involved in all the basic molecular functions including DNA binding, RNA binding, chromatin binding, protein binding, lipid biding, carbohydrate binding, transcription factor activity, transcription regulator activity, translation factor activity, translation regulator activity, signal transducer activity, transporter activity, kinase activity, transferase activity, hydrolase activity, enzyme regulator activity, and others. As shown in Fig. 3, each of these GO terms was enriched with a similar number of proteins from the two tomato lines. These results indicate that the quantified proteomes from the GlyI and ECtr root-tips are largely comprised of proteins in the same functional pathways to support the same biological and physiological processes. The quantified proteomes were then analyzed to identify Al-induced differentially expressed proteins (DEPs).

### Al-induced differentially expressed proteins (DEPs)

For the identification of DEPs in each tomato line, the Fold (T/C) values of the quantified proteins were tested for normal distribution using the Good-of-Fit algorithum. The Fold (T/C) values of the whole proteomes passed tests of Kolmogorov–Smirnov (Pr > D; *P* < 0.010), Cramer–von Mises (Pr > W-Sq, *P* < 0.005), and Anderson–Darling (Pr > A-Sq, *P* < 0.005). The value of two standard deviation (SD) of normal distribution was obtained for the proteome of each line. The Al-induced DEPs were listed by filtering each proteome using the following criteria: quantified with at least two unique peptides, *P* < 0.05 (calculated in PD 2.2 using a post hoc Tukey’s HSD test), and Fold (T/C) > 2 SD or < -2SD. From the GlyI line, 201 DEPs, with Fold (T/C) > 0.46, or <−0.46, were listed, which accounts for 4.9% of the quantified proteins. In the ECtr line, 230 DEPs, with Fold (T/C) > 0.48, or <−0.48, were identified, which represents 5.5% of the quantified proteins. In total, 431 DEPs were identified in the two tomato lines, 50 proteins found in both lines, and 381 DEPs were found only in one of the two lines (Tables S4 and S5).

**Fig. 4:**
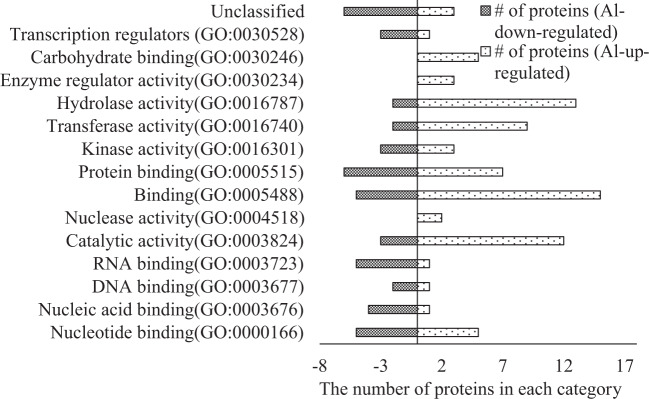
Categories of molecular function using Plant MetGenMap analysis of Al-induced differentially expressed proteins identified in both ECtr and GlyI lines. The GO term identification (ID) is provided in the bracket. On the *X*-axis, the negative numbers indicate Al-down-regulated proteins. Some proteins were placed in multiple categories

Among the 50 DEPs identified in both GlyI and ECtr lines, 49 proteins showing the same trend of changes from non-Al-treated to Al-treated conditions with 32 up-regulated and 17 down-regulated DEPs, only one protein was repressed in ECtr, but induced in GlyI. This group of DEPs were analyzed for functional classification GOs by Plant MetgenMap classification system. The 50 DEPs were classified into 15 molecular functional groups (GOs) (Fig. 4, Table S6). Proteins associated with transcription, translation (transcription regulator activity, RNA binding, DNA binding, nucleic acid binding) were all repressed under Al-treated conditions. Functional groups for catalytic activity, hydrolase activity and transferase activity contain more Al-up-regulated proteins than repressed proteins. Two functional groups for carbohydrate binding and enzyme regulator activity contain only Al-up-regulated proteins.

**Fig. 5:**
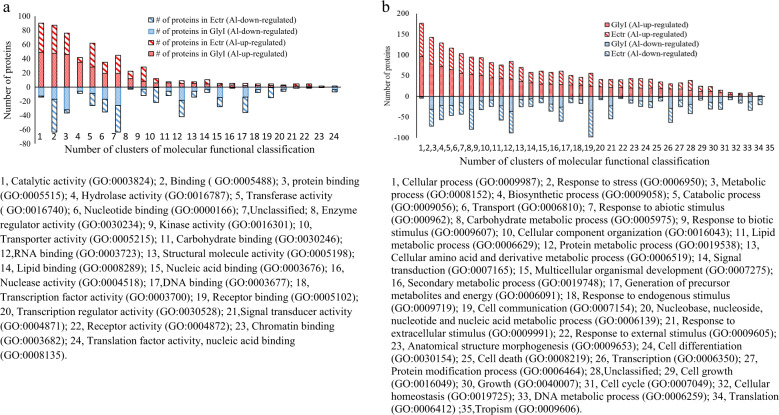
Categories of molecular function **a**, and biological process **b** using Plant MetGenMap analysis of Al-induced differentially expressed proteins identified in ECtr and GlyI lines. The GO term identification (ID) is provided in the bracket. On the *X*-axis, the negative numbers indicate Al-down-regulated proteins. Some proteins were placed in multiple categories

The DEPs identified from each individual line were classified based on molecular functional pathways and biological processes, and the number of proteins in each GO was compared between GlyI and ECtr lines (Fig. 5a, b; Table S8). According to molecular functions, these proteins were clustered into 24 GOs. For the Al-up-regulated DEPs, proteins from GlyI constituted eight clusters each comprising of a minimum of 12 proteins. These GOs include catalytic activity (cluster 1), binding (cluster 2), protein binding (cluster 3), hydrolase activity (cluster 4), transferase activity (cluster 5), nucleotide binding (cluster 6), enzyme regulator activity (cluster 7), and an unclassified group (cluster 8). In ECtr line, only seven proteins were enriched in the hydrolase activity group (cluster 4). For the Al-down-regulated DEPs, cluster 1 (catalytic activity) has 13 proteins in GlyI, but only one protein in ECtr; the receptor binding (cluster 19) group comprises of 14 DEPs in ECtr, but only one in GlyI. The transcription factor activity (cluster 18) and transcription regulator activity (cluster 20) were enriched with the same number of proteins from GlyI and ECtr lines. These results indicate that the Al-induced DEPs are associated with various molecular functions in both GlyI and ECtr lines.

The Al-induced DEPs were clustered into 35 GOs of biological processes (excluding those containing <5 proteins) (Fig. 3b). Biological processes containing a greater number of Al-up-regulated than the down-regulated proteins include response to stress (cluster 2), metabolic processes of carbohydrates, lipids and cellular amino acid and derivative, signal transduction, cell communication, cell death, and cell growth. Biological processes including the basic gene expression processes from transcription to translation (clusters 26, 34), cell cycle (cluster 31), tropism (cluster 35), and DNA metabolic process (cluster 33) were enriched with a larger number of Al-down-regulated than Al-up-regulated DEPs. The protein modification process (cluster 27) contains a larger number of Al-down-regulated but fewer Al-up-regulated DEPs in ECtr compared to GlyI. More importantly, when examined within the same GOs, some of the pathways were enriched with proteins of different identity from GlyI and ECtr, or the same proteins showing opposite Al-induced changes in the two lines.

### Al-induced proteins associated with MG and Al toxicity

Glyoxalase system is comprised of Gly I and Gly II. The relative abundance [Fold (T/C)] of the protein encoded by the inserted gene (Solyc09g082120.3.1) had no change in the GlyI line. The same protein accession showed some Al-induced decrease in ECtr line (20%). In GlyI, the *SlGlyI* gene was driven by 2 × 35S promoter, and the constitutive expression of the inserted gene should have produced proteins to compensate for the Al-induced decrease as seen in ECtr. Two additional proteins annotated to Gly I were identified in GlyI, they are Solyc02g080630.3.1 (0.51-fold, *p* = 0.01) and Solyc12g007310.2.1 (0.30-fold, *p* = 0.03). Two glyoxalase II proteins also increased in abundance under the Al-treated conditions in GlyI (Solyc06g053310.3.1, 0.41-fold; Solyc07g061960.3.1,0.40-fold; *p* = 0.03–0.04). None of these proteins showed significant changes in ECtr line (Table 1).

**Table 1:** The Al-induced differentially expressed proteins associated with degradation of methylglyoxal and Al toxicity in tomato plants overexpressing a *SlGlyI* gene

Biological function	Protein description	Fold (T/C)^a^	
		GlyI^b^	ECtr^c^
MG detoxification	Glyoxalase I (Solyc02g080630.3.1)	0.51*^d^	n/i^e^
	Glyoxalase I (Solyc12g007310.2.1)	0.36*^d^	0
	Glyoxalase I (Solyc07g040950.3.1)	−0.25	0.12
	Glyoxalase I (Solyc06g007610.2.1)	−0.1*^d^	−0.06
	Glyoxalase I (Solyc09g082120.3.1)^f^	0	−0.32
	Glyoxalase II (Solyc06g053310.3.1)	0.41*^d^	0.04
	Glyoxalase II (Solyc07g061960.3.1)	0.40*^d^	0.08
Callose degredation	Beta-1 3-glucanases (Solyc01g008620.3.1; Solyc01g060020.3.1)	0.72–0.76*^d^	n/i
Cell wall matrix: Xylan	Acetyl xylan esterase A (Solyc04g078440.3.1)	−0.60*^d^	n/i
	Xylanase inhibitor (Solyc01g080010.2.1)	n/i	0.53*^d^
	Caffeoyl-CoA O-methyltransferase (Solyc03g032220.3.1), O-methyltransferase 3 (Solyc10g008120.3.1)	0.74*^d^ 0.79*^d^	n/i
Lignin	Laccase-22 (Solyc08g079090.3.1)	−0.05	0.51*^d^
	Polygalacturonases (Solyc05g005170, Solyc09g075460.3.1) Polygalacturonase inhibitor (Solyc09g014480)	n/i	0.51–0.59*^d^ −0.67*^d^
	Glutathione S-transferases (6 enzymes)	0.66 –1.18*^d^	0.67–0.69*^d^
Pectin			
H_2_O_2_ metabolism	Peroxidases (4 enzymes)	0.54–0.73*^d^	0.55–0.71*^d^
	Polyphenol oxidase (Solyc02g078650.3.1)	0.54*^d^	n/i
	Oxalate oxidase (Solyc03g123410.1.1)	0.48*^d^	n/i
Vacolar sequestration	Citrate binding protein (Solyc11g005480.2.1)	0.78*^d^	n/i

^a^Log2 value of protein abundance ratio between Al-treated to non-Al-treated conditions reported in PD 2.2 analysis

^b^Tomato transgenic line overexpressing *SlGlyI* gene

^c^The positive control tomato line transformed with empty vector

^d^Al-induced differentially expressed proteins (DEPs) quantified with at least two unique peptides, with *P* < 0.05 in PD 2.2 using a post hoc Tukey’s HSD test, and Fold (T/C) greater than twice of standard deviation (SD) derived from normal distribution of the quantifed proteome

^e^Proteins not showing significant changes from Al-treated to non-Al-treated conditions, or not identified in the respective tomato line

^f^Protein accession encoded by the inserted *SlGlyI* gene

Additionally, several Al-induced DEPs are involved in biological process affecting plant responses to Al toxicity. These include callose metabolism associated with the plasmodesmata (Pd)-mediated cell–cell signaling, cell wall composition, modulation of the stress-induced oxidative stress, and possible Al exclusion mechanisms (Table 1). The beta-1,3-glucan synthase with a function in degrading callose on Pd was induced in Al-treated GlyI root-tips. The Al-induced DEPs affecting cell wall matrix include acetyl xylan esterase, laccase, galacturonase, and polygalacturonase inhibitor protein 1. Acetyl xylan esterase is an enzyme that catalyzes the deacetylation of xylans and xylo-oligosaccharides, and it was reduced in Al-treated GlyI. The CAD and O-methyltransferase 3 in the lignin biosynthesis pathway were increased in GlyI under Al-treated condition. For pectin metabolism, polygalacturonase (pectin depolymerase) was increased in ECtr, whereas the polygalacturonase inhibitor was induced in Al-treated GlyI. While H_2_O_2_-generating oxalate oxidase was increased only in GlyI, several ROS-detoxifying enzymes, such as glutathione-S-transferase (GST) and peroxidases were induced in GlyI and ECtr lines. A citrate-binding protein (Solyc11g005480.2.1) for vacuolar transport of citrate with a possible role in chelating Al^3+^ in apoplatic space was increased in GlyI (0.78-fold) under Al-treated condition.

### Network analysis of Al-induced differentially expressed proteins

**Fig. 6:**
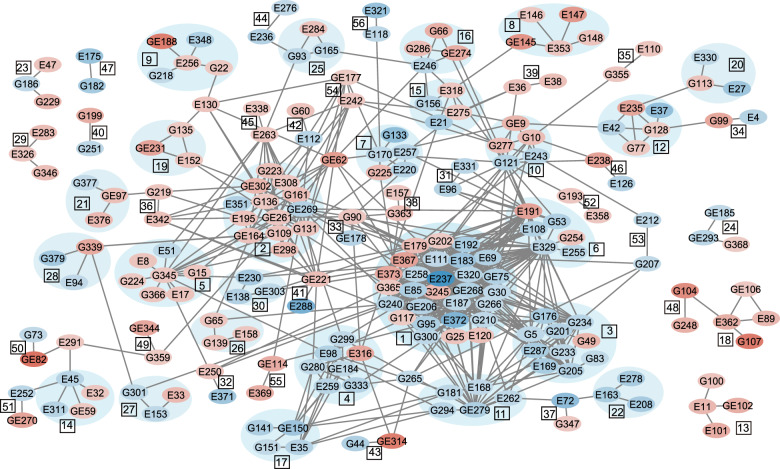
Cytoscape image of protein–protein interaction network constructed using STRING analysis performed on Al-induced differentially expressed proteins in either GlyI or ECtr line. Circles in red are Al-up-regulated proteins; Circles in blue are Al-down-regulated proteins. The depth of the color indicates the magnitude of Log2Fold change between Al-treated and non-Al-treated control groups with darker color correlates with bigger difference

As described above, the proteomics analysis has identified a list of DEPs in either GlyI or ECtr lines. These line-specific DEPs were used to construct an association network using the STRING V11.0 software (at 0.400 medium confidence level). Out of the 381 DEPs, 217 proteins constituted 60 clusters that form very complex and strongly interactive networks using the MCL with the inflation parameter 3 (MCL = 3) (Fig. S2, Table S8). According to the MCL, there are 665 edges, the average local clustering coefficient is 0.326, the expected number of edges is 500, and the PPI enrichment *p*-value: 1.42e−09. The network has significantly more interactions than expected, which indicates that these proteins are strongly related functionally or physically^54^. The Cytoscape network (Fig. 6) was constructed showing protein fold changes (blue means Al-down-regulated, red means Al-up-regulated) (Table S9). Cluster 1 proteins constituted ribosome (sly03010) and protein export (sly03060) KEGG pathways. Cluster 4 proteins formed the spliceosome and mRNA surveillance pathways. Cluster 3 proteins include CCAAT-binding transcription factor (G234), damaged DNA-binding 2 (E169), and rRNA-processing proteins (G5, E287,G176, G205,G201). Cluster 6 contains peptidases and peptidase inhibitors. Cluster 11 contains histone deacetylase HDT1 (E262), DNA-directed RNA polymerase III subunit RPC4 (E168), H/ACA ribonucleoprotein (GE279), and RNA-binding protein (G181, G294). Cluster 17 proteins are mRNA splicing factors. Cluster 28 proteins are involved in epigenetic regulation of gene expression, such as G339 (histone H3) and G379 (cytosine-5 DNA methyltransferase), in Gly I line. Cluster 22 and 37 contain histone proteins from both lines. These clusters are all involved in gene expression/protein biosynthesis processes, and a majority of these proteins belong to Al-down-regulated DEPs.

Clusters comprising only Al-up-regulated DEPs are mainly involved in ubiquitin complex subunits in cluster 27, ABA-induced proteins (E147, ASR1;GE145,ASR2; E146,ASR3) in cluster 8, heat shock proteins serving as chaperones for ER protein processing in cluster 5, MG and Al detoxification (G65), lactoylglutathione lyase (G139), S-adenosylmethionine-dependent methyltransferase (E158) in cluster 26, Kunitz trypsin inhibitors (cluster 18), and cluster 13 (E101, GE102 for two PLAT domain-containing protein 1). Cluster 2 is comprised of proteins involved in glycolysis pathways, biosynthesis of secondary metabolites, and pyruvate metabolism. These proteins were mostly increased under Al-treated condition. Three proteins of GlyI line in cluster 10 (G277, GE9, G10) formed the oxidative phosphorylation KEGG pathway. In ECtr, the Al-down-regulated cytochrome b-c1 complex subunit 7 (E243) is a member of electron transfer complex and plays a critical role in biochemical generation of ATP. These Al-induced DEPs affect the carbohydrate metabolic processes under Al-treated conditions. Using the information of proteins in the same network but expressing differential Al-induced changes between GlyI and ECtr lines, we can identify those that are associated with the over-expression of the glyoxalase I gene.

## Discussion

Plants, when exposed to Al^3+^ ions, experience direct injury from the toxic ions as well as secondary physiological stresses including accumulation of many toxic metaoblites, such as reactive oxygen species (ROS), MG, and others^57^. The stress-induced overproduction of MG can cause alteration in gene expression, disruption of normal cellular functions and metabolic pathways and, in some instances the interference in signal transduction pathways due to protein modification can lead to cell death or arrest of growth^16,58,59^. Thus the accumulation of the MG exacerbates the cellular damages from Al toxicity.

Lactoylglutathione lyase/GlyI is a critical enzyme that can determine the rate of MG detoxification^9,60^. In this study, tomato transgenic lines overexpressing a tomato glyoxalase I gene were produced. Physiological analysis showed that these plants expressed significantly higher Gly I activity, enhanced the capacity of the MG-detoxifying glyoxalase pathway by increasing the protein abundance of Gly I and II, and lowered MG levels. In the GSH-dependent glyoxalase pathway, Gly I is the first enzyme which catalyzes the isomerization of MG into S-2-hydroxyacylglutathione as the substrate of Gly II^58,61^. Therefore, activation of Gly I is the first and critical step for the MG detoxification system^21,62^. The quantitative proteomics analysis revealed that in the GlyI line, in addition to the protein encoded by the inserted gene, additional isoforms of Gly I and Gly II were induced under Al-treated condition, which led to an increase in the activity of glyoxalase pathway. These results suggest a mechanism to co-regulate some of the Gly I isoforms in the tomato genome, which needs to be investigated in future studies.

Plants utilize two different mechanisms to develop Al tolerance: the Al exclusion to prevent/reduce the entry of the ions into root-tip cells and the ability to tolerate internalized Al^63,64^. Hematoxylin is a dye which forms a complex with Al, and the color intensity of stained root apices can be used as a measurement of the amount of Al accumulated/internalized in root-tip cells^43,44^. In this study, the color intensity of hematoxylin-stained root-tips from GlyI and ECtr plants was very similar, and both had radial swollen root-tips which is a major symptom of Al toxicity^65^. Based on these results, we concluded that the GlyI plants did not have significant improvement on reducing the amount of Al uptake into root-tips. As described above, the GlyI-overexpressing plants were able to lower the MG content level which suggests that the associated molecular mechanisms in GlyI rely on tolerance to internalized Al^3+^ and the induced secondary cellular stress factors.

According to the proteomics analysis and the molecular functions of Al-induced DEPs, the Gly I line increased capacity in the following biological processes that are related to Al toxicity: callose metabolism affecting the plasmodesmata (Pd)-mediated cell–cell signaling, cell wall matrix and oxidative stress, and repair of DNA damages. The Al-induced callose accumulation on Pd can slow-down or completely stop trafficking of molecules intercellularly, which was reported to cause stoppage of root elongation under the stress condition^66,67^. The Al-induced beta-1,3-glucan synthase protein in GlyI can increase the capacity of degrading callose at Pd^68^. The higher abundance of this protein might provide a mechanism for maintaining cell–cell exchanges in signaling molecules and metabolites under Al-treated condition.

The second mechanism is associated with plant cell wall. Plant cell wall matrix is comprised of cellulose, hemicellulose, lignin, and pectin components. Previous studies showed that hemicellulose is the major Al-binding component^69^. Xyloglucan (XyG) is the principal hemicellulose in primary walls of dicots, where a *O*-acetylated XyG backbone is present in Solanaceae as well as in grass^70^. The acetylation of XyG affects its Al^3+^-binding capacity^71^, as lowering acetylation level makes plants more sensitive to Al treamtents^72^. Acetyl xylan esterase catalyzes the reaction of deacetylation of substituted xylans^73^. In GlyI, the acetyl xylan esterase was reduced under Al-treated condition, which might have a role in maintaining the acetylation status of cell wall by suppressing the xylan deacetylation level and thus reducing senstitiy of roots to Al ions. Furthermore, the polygalacturonase inhibitor was decreased in GlyI line, this protein was shown to increase plant tolerance to protons (generated in low pH) under Al^3+^ toxic conditions^74^.

The third mechanism involves oxidative stress and displacement of the Al-injured epidermal cells. Both Al^3+^ toxicity and MG accumulation induce oxidative stress. The overexpression of ROS-detoxifying enzymes such as GST and peroxidases enhances Al resistance in several plant species^72,75,76^. Under Al-treated condition, these enzymes increased significantly in both GlyI and ECtr lines, but a greater number of DEPS were identified in GlyI line. Previous studies indicate that Al treatments induced H_2_O_2_-generating oxalate oxidase at transcript, protein level, and enzyme activity levels^31,77^. Subsequent accumulation of oxidative stress and cell death would accelerate the turn over of epidermal cells, and the replacement of these cells at root surface can protect deeper cell layers in the meristematic and elongation zones from the Al-induced injuries. Several plants were reported to utilize this mechanism to maintain root growth against Al toxicity^78^. The oxalate oxidase was induced in GlyI (0.48-fold) which can serve a similar function in tomato roots.

The fourth mechanism involves DNA repair. Excessive levels of MG cause DNA mutation and non-enzymatic glycation of proteins. Aluminum ion toxicity also causes damages to DNA and affects normal cell cycle^79^. While Al can induce MG accumulation, the latter also exaggerates the injuries to plant cells from Al^3+^ toxicity. Among the Al-induced DEPs, the Damaged DNA-binding 2 (DDB2) (Solyc05g025900.3.1) was reduced in ECtr (−0.69-fold, *P* = 0.00), it was not changed in GlyI line (−0.09-fold, *P* = 0.54). DDB2 has a high affinity toward UV-damaged DNA; this enzyme has a key role in cellular activity of global genome nucleotide excision repair. Previous research reported that the DDB2 and associated partners have an important role in maintaining genome integrity under stress conditions^80,81^. In the STRING network, this protein (E169) is interconnected with ribosomal proteins (in cluster 3) and the histone deacetylase HDT1 (E262) with a function in repressing gene transcription (in cluster 11) and DNA-directed RNA polymerase III subunit RPC4 (E168) (in cluster 11) in ECtr line. These clusters were both enriched with Al-down-regulated DEPs. These results suggest that overexpression of GlyI may have some influence on protection of proteins which are involved in maintaining genome integrity when plants are subjected to Al^3+^ toxicity.

In conclusion, Al treatments induced MG accumulation in tomato root-tips, and inhibited tomato plant growth. The overexpression of the *SlGlyI* gene led to an increase in the glyoxalase system at protein and enzyme levels, and reduced the MG content under Al-treated conditions. The overexpression of *SlGlyI* gene helped to ameliorate, but not totally overcome, the Al-induced inhibitory effects on root growth from Al treatments. The global proteomics analysis identified that molecular processes of gene transcription and protein translation were constituted with Al-down-regulated proteins which concurs with our previous proteomics studies in tomato and switchgrass. According to the analysis of the Al-induced DEPs in GlyI, the transgenic plants have enhanced molecular functions and biological processes to ameliorate the injuries from Al^3+^ and MG toxicity. In this study, we have constructed a network for DEPs identified from GlyI and ECtr lines. Using the map, we can visualize differentially expressed proteins in either line within the same pathways or those connecting different clusters and/or pathways.

## Supplementary information

Fig. S1 Validation of insert gene sequence in transgenic tomato plants

Fig. S2 String image of protein-protein interactions of Al-induced differentially expressed proteins in GlyI and ECtr lines

Table S1. TMT-proteomics report and quantified proteins in SlGlyI-overexpressing tomato line GlyI

Table S2. TMT-proteomics report and quantified proteins in ECtr tomato line

Table S3. Molecular function Gene Ontology (GO) classification of quantified proteins from GlyI and ECtr tomato lines

Table S4 Al-induced differentially expressed proteins in GlyI for Plant MetGen Map analysis.

Table S5 Al-induced differentially expressed proteins in ECtr for Plant MetGen Map analysis.

Table S6 Al-induced differentially expresssed proteins common in GlyI and ECtr lines

Table S7 List of Al-induced differential expressed proteins used in Plant MetGen Map analysis

Table S8 Proteins and MCL clusters for STRING Protein-protein interaction network

Table S9 List of proteins and interactions in Cytoscape image
