# Supplementary material for: Al-induced proteomics changes in tomato plants over-expressing a glyoxalase I gene
Source: Hortic Res. 2020 Apr 1;7:43. doi: 10.1038/s41438-020-0264-x (PMC7109090; doi:10.1038/s41438-020-0264-x)
Supplement: Supplementary file 1 — Fig. S1 Validation of insert gene sequence in transgenic tomato plants [file 41438_2020_264_MOESM1_ESM.pdf]

Fig. S1. Validation of insert gene sequence in transgenic tomato plants

DNA samples extracted from plant leaves were used in PCR reactions using two pairs of primers: 35S forward and gene-specific reverse primers for *SlGlY1* and primers for the BAR gene (35SF: CTATCCTTCGCAAGACCCTTC, *SlGlY1*R: TAGGAGGCAGGAGCCCCA, BarF-5'-AGTCGACCGTGTACGTCTCC-3', BarR-3'-GAAGTCCAGCTGCCAGAAAC-5'). The PCR bands were cloned onto T/A cloning vector and plasmids were sequenced using T7 sequencing primer at GenHunter (Nashville, TN). The *SlGlY1* insert sequence was translated into peptides online at https://web.expasy.org/translate/ and the peptide was blasted in Tomato Genome Protein database (ITAG release 3.20). The insert sequence matched tomato accession Solyc09g082120.2. The bar gene sequence was searched in NCBI database. The insert DNA sequences are:

>SlGlyI
TTCTCAACACAACATATACAAAACAAACGAATCTCAAGCAATCAAGCATTCTACTTC
TATTGCAGCAATTTAAATCATTTCTTTTAAAGCAAAAGCAATTTTCTGAAAATTTTCA
CCATTTACGAACGATAGCCATGGTCCGGACTCAGATCTCGAGATGGCAGCTCAAGG
AACTTGCCTTAATCACGTCTCCAGAGAATCCTCTGATATCAAACGCCTAGCCCAATT
TTACATTGAGATATTTGGGTTTCAGAAAGTTGAAGCTCCAAGATTTGAATTTGATGT
GATATGGTTGAAGCTGGCACCGTCCTTTTATCTTCACCTTATCGAGAGGGACCCGAC
TACGAAGCTTCCAGAAGGTCCATGGAGTGCCACGTCAGCCATCGCTGACCCGAAGA
ATCTGCCCAGAGGTCACCATGTCTGCTTCACCATCTCCAATTTCGATTCCTTTGTTCA
GAAGCTCAAGGATAAAGGAATTGAAGTTCATGAGAGGACTCAACCAGATGGCAAGA
CCAAACAAGCCTTTTTCTTTGATCCAGATGGAAATGGTTTGGAGGTAAGCAGTGGGG
CTCCTGCCTCCTAATCGAATTCCCGCGGCCGCC

>Bar
NNNNNNNNNNNANGNTCCNGCCGCCNTGGCGGCCGCGGGAATTCGATTGAAGTCCA
GCTGCCAGAAACCCACGTCATGCCAGTTCCCGTGCTTGAAGCCGGCCGCCCGCAGC
ATGCCGCGGGGGGCATATCCGAGCGCCTCGTGCATGCGCACGCTCGGGTCGTTGGG
CAGCCCGATGACAGCGACCACGCTCTTGAAGCCCTGTGCCTCCAGGGACTTCAGCA
GGTGGGTGTAGAGCGTGGAGCCCAGTCCCGTCCGCTGGTGGCGGGGGGAGACGTAC
ACGGTCGACCAATCACTAGTGAATTCGCGGCCGCCTGCAGGTCGACCATATGGGAG
AGCTCCCAACGCGTTGGATGCATAGCTTGAGTATTCTATAGTGTCACCTAAATAGCT
TGGCGTAATCATGGTCATAGCTGTTTCCTGTGTGAAATTGTTATCCGCTCA

Residues 1 - 775 775 residues shown,

```
>Solyc09g082120.2 Glyoxalase/bleomycin resistance protein/dioxygenase (AHRD V1 ***- D1C786_SPHTD); contains Interpro d
IPR004360 Glyoxalase/bleomycin resistance protein/dioxygenase BLAST
CATTTAAATG GGTAGCAAAA TTCTACTTGA ATTCTCTTCA ACACAAACAA GAACAAAAAT GGCAGCTCAA GGAACTTGCC TTAATCACGT CTCCAGAGAA
TCCTCTGATA TCAAACGCCT AGCCCAATTT TACATTGAGA TATTTGGGTT TCAGAAAGTT GAAGCTCCAA GATTTGAATT TGATGTGATA TGGTTGAAGC
TGGCACCGTC CTTTTATCTT CACCTTATCG AGAGAGACCC GACTACGAAG CTTCCAGAAG GTCCATGGAG TGCCACGTCA GCCATCGCTG ACCCGAAGAA
TCTGCCCAGA GGTCACCATG TCTGCTTCAC CATCTCCAAT TTCGATTCCT TTGTTCAGAA GCTCAAGGAT AACGGAATTG AAGTTCATGA GAGGACTCAA
CCGGATGGCA AGACCAAACA AGCCTTTTTC TTTGATCCAG ATGGAAATGG TTTGGAGGTA AGCAGTGGGG CTCCTGCCTC CTAGACAGTA GTGACCAAAC
TCTTGGTGTT CCGCGTAAGT TTTAGAGTAT AATAAACCCA AAGGACTCGA AAAACATTAG CTCGAATGAT GACCTTGTGT AAATTTTCGT ATTGGTATGA
AAATATTGTG TCGTTTAGCT ATGCTTTCTC CTTGCAATTG CCTCCAAGAA ACTTGTACCC TGGTTGTTAC CTATACTCCC TTCGTCCGGT ACTGGTTTCT
ATTTTCTATT TTTTGAGTCA AACTATAAAA ATTTTGGGTA ACATTTTAAG ATGAATTTTT TCCTCATATT AATAT
```

Download ⌄ GenBank Graphics

Synthetic construct clone pPK2-BAR BAR (bar) gene, complete cds

Sequence ID: MF169981.1 Length: 1758 Number of Matches: 1

Range 1: 622 to 798 GenBank Graphics ▼ Next Match

Score	Expect	Identities	Gaps	Stran
327 bits(177)	6e-86	177/177(100%)	0/177(0%)	Plus/P

```
Query  1    ATGCCGCGGGGGGCATATCCGAGCGCCTCGTGCATGCGCACGCTCGGGTCGTTGGGCAGC  60
            ||||||||||||||||||||||||||||||||||||||||||||||||||||||||||||
Sbjct  622  ATGCCGCGGGGGGCATATCCGAGCGCCTCGTGCATGCGCACGCTCGGGTCGTTGGGCAGC  681

Query  61   CCGATGACAGCGACCACGCTCTTGAAGCCCTGTGCCTCCAGGGACTTCAGCAGGTGGGTG  120
            ||||||||||||||||||||||||||||||||||||||||||||||||||||||||||||
Sbjct  682  CCGATGACAGCGACCACGCTCTTGAAGCCCTGTGCCTCCAGGGACTTCAGCAGGTGGGTG  741

Query  121  TAGAGCGTGGAGCCCAGTCCCGTCCGCTGGTGGCGGGGGGAGACGTACACGGTCGAC  177
            |||||||||||||||||||||||||||||||||||||||||||||||||||||||||
Sbjct  742  TAGAGCGTGGAGCCCAGTCCCGTCCGCTGGTGGCGGGGGGAGACGTACACGGTCGAC  798
```
