# Supplementary figures and images for: Al-induced proteomics changes in tomato plants over-expressing a glyoxalase I gene
Source: Hortic Res. 2020 Apr 1;7:43. doi: 10.1038/s41438-020-0264-x (PMC7109090; doi:10.1038/s41438-020-0264-x)

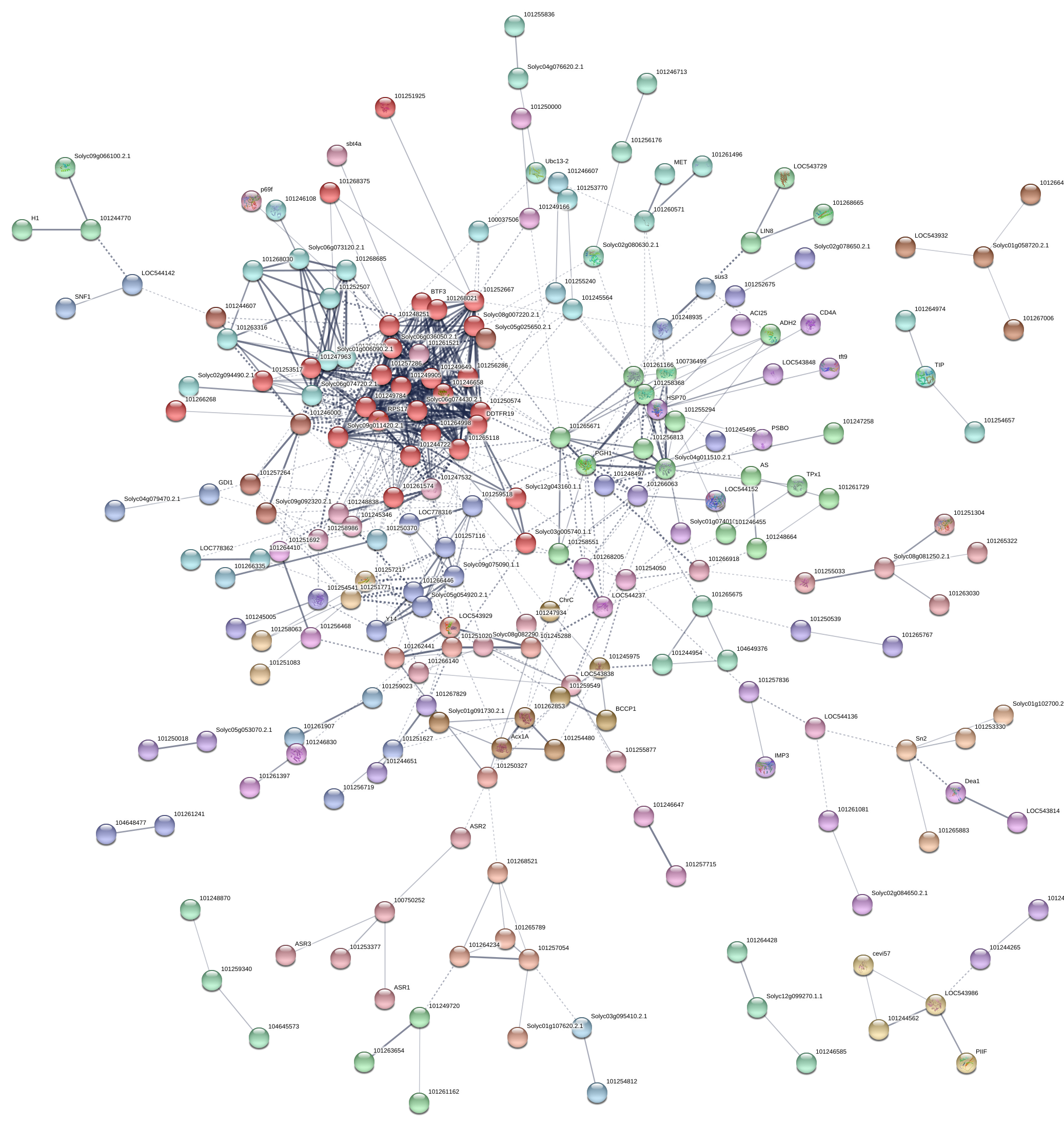

Supplement: Supplementary file 2 — Fig. S2 String image of protein-protein interactions of Al-induced differentially expressed proteins in GlyI and ECtr lines [file 41438_2020_264_MOESM2_ESM.png]
